# Calcium dysregulation in diabetic cardiomyopathy & heart failure with preserved ejection fraction

**DOI:** 10.3389/fcvm.2026.1872513

**Published:** 2026-06-23

**Authors:** Anza Ali, Vineet M. Sharma, Yuriana Aguilar-Sanchez

**Affiliations:** 1 Department of Veterinary Physiology and Pharmacology, College of Veterinary Medicine & Biomedical Sciences, Texas A&M University, College Station, TX, United States; 2 Department of Biomedical Engineering, College of Engineering, Texas A&M University, College Station, TX, United States

**Keywords:** Ca^2+^ dysregulation, Ca^2+^ handling, diabetic cardiomyopathy, gene therapy, HFpEF

## Abstract

Diabetes is a major risk factor for cardiovascular disease, and heart failure (HF), particularly heart failure with preserved ejection fraction (HFpEF), which is the most prevalent form of HF in patients with type 2 diabetes (T2D). However, the mechanisms behind diabetes-induced cardiomyopathy (DbCM) are complex and remain poorly understood. Insulin resistance resulting from diabetes has been shown to contribute to cardiac impairment, leading to diastolic dysfunction and hypertrophy. Studies have shown that the impairment of calcium (Ca^2+^)-handling cardiac proteins, such as sarcoplasmic reticulum Ca^2+^-ATPase (SERCA2a) and ryanodine receptor type 2 (RyR2), may act as key drivers behind the development of DbCM and its progression to HF. However, further studies are needed to fully understand their impact. This review focuses on the intersection of diabetes and HFpEF at the molecular level, showing how insulin resistance contributes to cardiac impairment, and the critical role of Ca^2+^ -handling proteins in DbCM progression. Due to the limited understanding and the complexity of DbCM, there are currently no viable cures that reverse disease progression in DbCM or HFpEF. However, adeno-associated virus (AAV)- mediated gene therapies show promise for treating diabetes-induced cardiomyopathy. This review discusses molecular pathways affected under DbCM and HFpEF conditions as well as potential treatments in both preclinical and clinical trials to analyze their effectiveness.

## 1 Introduction

**Figure 1:**
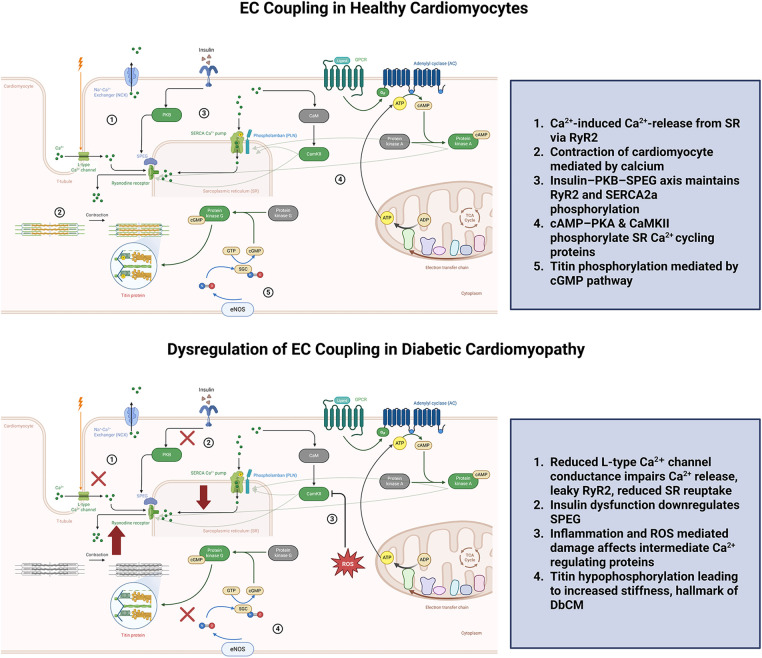
EC coupling in healthy cardiomyocytes and its dysregulation in diabetic cardiomyopathy. In healthy cardiomyocytes, action potential-triggered Ca^2^⁺ entry via L-type Ca^2^⁺ channels initiates calcium-induced calcium release from the SR through RyR2, driving contraction. The insulin–PKB–SPEG axis maintains RyR2 and SERCA2a phosphorylation, while cAMP–PKA–CaMKII signalling regulates key Ca^2^⁺ handling proteins and the cGMP pathway governs titin-mediated myofilament compliance. In diabetic cardiomyopathy, reduced L-type channel conductance, leaky RyR2, and impaired SR Ca^2^⁺ reuptake disrupt Ca^2^⁺ homeostasis. Insulin dysfunction suppresses PKB–SPEG signalling, mitochondria-derived ROS impairs intermediate Ca^2^⁺-regulating proteins, and titin hypophosphorylation increases myocardial stiffness which is a hallmark of DbCM. Created in BioRender. Sharma, V. (2026) https://BioRender.com/zgorvb3.

Diabetes is a chronic disease and one of the leading causes of cardiovascular disease and heart failure (HF). The World Health Organization (WHO) reported that in 2021, approximately 11% of all cardiovascular deaths were due to high or uncontrolled blood glucose^1^. The International Diabetes Federation reported that 589 million people aged 20–79 years worldwide, or roughly 1 out of 9 adults, live with diabetes^2^. Around 90%–95% of these patients are diagnosed with type 2 diabetes (T2D). The remaining 5%–10% of patients are diagnosed with either type 1 diabetes (T1D), gestational, or latent autoimmune diabetes (1). Despite differing etiologies, all forms of diabetes lead to increased rates of cardiovascular disease. Diabetic cardiomyopathy (DbCM) is characterized by cardiac dysfunction that occurs in the absence of significant coronary artery disease, hypertension, or valvular heart disease (2). DbCM is often underdiagnosed and can lead to HF, affecting both diastolic and systolic function. Given that HF involves impaired cardiac contraction, Ca^2+^ mishandling plays a critical role at the molecular level. Ca^2+^ handling governs excitation-contraction coupling (ECC) in the heart and relies on cardiac proteins like sarcoplasmic reticulum Ca^2+^-ATPase (SERCA2a), which reuptake Ca^2+^ into the sarcoplasmic reticulum (SR) and has shown to be impaired in patients with T2D diabetes. Figure 1 illustrates physiological Ca^2+^ handling mechanisms in healthy cardiomyocytes that are disrupted under pathological conditions like DbCM. Since Ca^2+^ is integral for proper cardiac function and due to the interconnection between diabetes and HF, treatments often need to target both diseases at the molecular level.

In this review, we discuss Ca^2+^ signaling dynamics under physiological conditions necessary for proper heart function and analyze diabetes as a driving factor of HF. We also discuss the underlying pathophysiological changes that occur as DbCM progresses from diastolic to systolic HF, and evaluate the effectiveness of current therapies that distinctly aim to treat different stages of DbCM progression.

## 2 Diabetes

Diabetes mellitus is characterized by either the body's inability to produce insulin (T1D) or the development of insulin resistance, which leads to the inability of the body to utilize insulin (T2D). According to the CDC's National Diabetes Statistics Report, in the U.S. alone, around 40.1 million people were diagnosed with diabetes, representing approximately 12% of the population. In addition, around 115.2 million U.S. adults had prediabetes, representing approximately 33%–34% of the population. Among the 40.1 million diagnosed, around 2.1 million had T1D, and the rest were diagnosed with T2D^3^. The American Diabetes Association (ADA) defines diagnostic criteria in which glycated hemoglobin A1c (HbA1c) ≥ 6.5%, fasting plasma glucose (FPG) ≥ 126 mg/dL, and random plasma glucose ≥ 200 mg/dL are thresholds for diabetes (3). T1D is characterized by hyperglycemia due to complete insulin deficiency. In T1D, the body's immune response destroys insulin-producing beta cells in the islets of Langerhans (4). Loss of insulin in T1D can be rapid or gradual, depending on various etiological factors such as genetic predisposition, environmental factors like exposure to viruses, and autoimmunity (5). T2D is characterized by insulin resistance or delayed insulin secretion (6). This can be caused by abnormalities in insulin signaling for glucose uptake, hereditary factors affecting beta-cell function, obesity that leads to insulin insensitivity, and disrupted molecular circadian rhythms (7). These variations in the disease foster different associated mechanisms where T1D is more associated with oxidative stress and T2D with lipotoxicity. The underlying mechanisms of T2D make it prone to cultivating chronic hyperinsulinemia, which alters cardiac insulin signaling and increases the risk of developing DbCM. Prediabetes is characterized by impaired glucose tolerance (IGT) and impaired fasting glucose (IFG) levels (8). Glucose readings are elevated but do not cross the threshold of hyperglycemia recommended by the ADA (7). Fasting glucose is ≥ 100 mg/dL but ≤ 126 mg/dL, and glucose tolerance test (GTT) readings are ≥ 140 mg/dL but ≤ 200 mg/dL (3).

### 2.1 Mechanisms of insulin resistance and metabolic dysfunction

Diabetes can lead to high blood glucose levels due to impaired glucose metabolism, which can cause serious damage to nerves and blood vessels, inducing retinopathy, kidney failure, cardiomyopathy, and limb amputations. Glucose metabolism is tightly regulated, with beta cells producing insulin that signals a myriad of metabolic processes. Under normal circumstances these include insulin-stimulated glucose uptake through glucose transporter type 4 (GLUT4) translocation in skeletal muscle and enhanced protein synthesis. In the liver, insulin suppresses gluconeogenesis and stimulates triglyceride formation and in adipose tissue, it suppresses lipolysis and reduces fatty acid release to the liver (9, 10). For decades, insulin resistance has been considered one of the major hallmarks of T2D (11). In the case of insulin resistance, target cells in adipose tissue, skeletal muscle, and the liver develop reduced sensitivity to insulin signaling (12). Obesity has been strongly linked to insulin resistance, as it leads to the accumulation of excess saturated fatty acids and diacylglycerols intracellularly blocking the PI3K-Akt signaling that leads to reduced glucose uptake (9). As well as ectopic lipid deposition extracellularly, that contributes to the local lipotoxic stress and inflammation which impairs the normal insulin pathways reducing insulin signaling (9). Studies have shown that insulin resistance leads to beta cell dysfunction resulting in hyperglycemia (13). Redox or reactive oxygen species (ROS) modifications alter beta cell functions, which leads to hyperinsulinemia causing insulin resistance as an adaptive response to maintain normal levels of fat and glucose until beta cells become exhausted and collapse (14, 15). Conversely, recent literature suggests that hyperinsulinemia might be the key driver that leads to obesity, which then forges insulin resistance and ultimately beta cell dysfunction. Strong evidence has also emerged showing that inhibiting hyperinsulinemia leads to weight loss, further solidifying it as the root cause of insulin resistance and not the other way around (16–18). It has turned into a debate whether hyperinsulinemia precedes insulin resistance or vice versa. Recent literature suggests that instead of seeing hyperinsulinemia as a unidirectional consequence of insulin resistance, it should be viewed as a driving force that initiates and worsens insulin resistance over time. In other words, hyperinsulinemia is now considered to have both a causative and compensatory role depending on the progression of diabetes and tissue location, not just one or the other.

## 3 Heart failure

HF can be classified into the following groups: heart failure with preserved ejection fraction (HFpEF), heart failure with reduced ejection fraction (HFrEF), heart failure with mildly reduced ejection fraction (HFmrEF), and right-sided HF. HFpEF has been primarily characterized by diastolic dysfunction, where left ventricular ejection fraction (LVEF) remains > 50%, but symptoms can worsen with associated comorbidities and in the absence of intervention (19). Diastolic function is mainly regulated by SERCA2a, which facilitates the Ca^2+^ reuptake into the SR, causing the relaxation of the ventricles (20). Underlying pathological conditions like diabetes and obesity can lead to left ventricular (LV) hypertrophy, increased atrial filling pressures, and Ca^2+^ mishandling due to SERCA2a structural domains dysfunction (21). The progression of HFpEF is classified into two stages, during the first stage, the disease progresses asymptomatically, and an increase in fibrosis can be observed (22). This results in cardiomyocyte stiffness and hypertrophy, leading to impaired filling of the left ventricle. Minor alterations in diastolic filling and atrial volumes appear; this is induced by the underlying pathological factors such as hyperglycemia and cardiac insulin resistance, which impair Ca^2+^ release and reuptake in cardiomyocytes (22). As the disease progresses, it enters the second stage, where the left ventricle becomes hypertrophic, diastolic dysfunction worsens, and symptoms such as shortness of breath, fatigue, irregular heartbeat, chest pain, and lightheadedness or fainting start to appear (22). Recent studies have shown HFpEF, HFmrEF and HFrEF as different manifestations or severity of HF with distinct cellular and structural remodelling but oftentimes shared mechanistic drivers (23). HFpEF sustains t-tubular structures and normal Ca^2+^ transients during early stages of the disease enabling preserved EF longitudinally with a gradual decline as compared to HFrEF (24–26). HFrEF patients were observed to undergo severe cardiac tissue remodeling including LV hypertrophy, which leads to impaired contractility over time (27, 28). Sequential transitions from HFpEF to HFrEF are rare but happen under superimposed cardiac injury (23).

### 3.1 Hallmarks and diagnostic characteristics of HFpEF

Sex differences are integral to HFpEF, and the disease disproportionately affects post menopausal women (29). Ventricular stiffness, microvascular dysfunction, and adipose distribution all contribute; however, the diagnostic criteria of the disease fails to account for these differences. It has been shown that women exhibit distinct remodeling patterns, concentric hypertrophy, and biomarker profiles that complicate the diagnosis (30). Women account for almost 50%–60% of clinical trial cohorts for HFpEF as compared to HFrEF where women account for 20%–25% of the cohort (31–35). This sex-based predominance is a distinguishing factor of HFpEF. Despite HFpEF being prevalent in women, well established HFpEF animal models majorly consist of male species (28). This is because it has been difficult to replicate female murine models to study HFpEF as female mice have higher baseline estrogen levels which do not decline rapidly as seen in humans. This makes female mice less susceptible to HFpEF-induced LV stiffening (36).

HFpEF is mainly characterized by diastolic dysfunction that is driven by numerous factors including calcium mishandling, compromised pathways, vascular and inflammatory drivers (37–40). Calcium dysregulation is a shared driver between HFrEF and HFpEF but has distinct mechanisms at play in the progression of both diseases. In HFrEF quantitative or reduced expression of calcium handling proteins like SERCA2a are widely observed whereas in diastolic dysfunction alterations are more localized (20, 41). Such as in DbCM or T2D models alterations in the cAMP signaling driven by localized remodeling of *β*-adrenergic receptors within the phospholamban (PLN) –SERCA2a microdomain were detected (21). A shift was observed in *db/db* mice from *β*1 to *β*2 -adrenergic receptor control as a compensatory mechanism, when *β*1-adrenergic receptor responses were blunted leading to impaired relaxation (21). PLN serves as a primary inhibitor of SERCA2a suppressing Ca^2+^ reuptake into SR when unphosphorylated (42). Under pathological conditions like HFpEF impaired *β*1-adrenergic receptors lead to disruption of the PLN-SERCA2a axis leading to slower Ca^2+^ decay and elevated diastolic Ca^2+^ levels progressively (21, 43). Similarly, RyR2 receptors become progressively hypersensitive in HFpEF models majorly affected by exercise or stress triggered instability leading to arrhythmogenesis whereas the Ca^2+^ SR load remains closer to normal contrary to HFrEF phenotypes where aggressive hyperphosphorylation of RyR2 channels leads to extensive SR depletion (44, 45). HFpEF is often accompanied by non-cardiac comorbidities like renal dysfunction, obesity, and hypertension, which further impair cardiac function, and increase metabolic stress on the body (46, 47). HFpEF accounts for approximately 50% of HF cases, while 45%–50% is attributed to HFrEF while the remaining 5%–10% are accounted for by HFmrEF (48). Despite its high prevalence, there is limited knowledge about the intracellular Ca^2+^ dynamics and mishandling involved in HFpEF progression compared to HFrEF (49). This is because there is no independent gold standard for diagnosing HFpEF when considering its mechanisms and phenotypes. Factors like age, obesity, and loading conditions contribute to the diagnostic criteria for HFpEF, not just true diastolic dysfunction. The set parameters that define HFpEF, such as ejection fraction (EF) ≥ 50% are load and method-dependent and often fail to capture the systolic impairment present. The lack of structural characteristics makes it difficult to produce a definitive diagnosis, as usually seen in HFrEF. HFpEF is also associated with multiple comorbidities, and is typically not a standalone disease, which further complicates the tracking of disease progression and treatment. Although studies in murine models have shown that diastolic dysfunction may arise through Ca^2+^ impairment, there are differences in the Ca^2+^ regulation between HFpEF and HFrEF (19, 21). HFpEF is a complex disease with several factors affecting prognosis, it is difficult to pinpoint which cardiac molecular pathway is impaired and which comorbidity contributes to the impairment. Since HFpEF is consequential to multiple pathological factors, treatment options are limited, and the present ones only slow down disease progression rather than act as a cure or reversal (50).

## 4 Diabetic cardiomyopathy induced HFpEF

In addition to chronic metabolic dysfunction, unmanaged diabetes mellitus can lead to DbCM, even in the absence of other cardiovascular risk factors (51). DbCM is characterized by inflammation, fibrosis, and insulin resistance, which leads to altered cardiac metabolic homeostasis and progressive myocardial impairment (51). Individuals with diabetes are twice as likely to develop heart failure (HF) or cardiovascular diseases, and recent data from 2021 indicates that approximately 1.78 million individuals with diabetes were hospitalized due to major cardiovascular diseases^4^ (52). Factors such as obesity, chronic inflammation, elevated triglyceride levels, unhealthy diets, alcohol consumption that manifest into high blood pressure, insulin resistance, and hyperglycemia predispose individuals with diabetes to cardiovascular diseases (53). Patients with diabetes and uncontrolled baseline glycated hemoglobin (HbA1c) levels below 6.2% or above 7.6% reflecting inadequate glycemic control, are at higher risk of all-cause mortality among individuals with both diabetes and cardiovascular diseases (54).

Although HbA1c levels have been used to assess hyperglycemia and cardiac risk, they do not capture key metabolic drivers of cardiac comorbidities, such as lipotoxicity, insulin resistance, and vascular inflammation. Chronic high glucose levels lead to the formation of advanced glycation end-products (AGEs), which contribute to oxidative stress and promote fibrosis and hypertrophy through interaction with receptor for advanced glycation endproducts (RAGE) receptors on cardiomyocytes (55). Myocardial lipotoxicity, a manifestation of chronic metabolic dysfunction, is another major contributor that increases fatty acid uptake and reduces glucose uptake in cardiomyocytes, leading to triglyceride accumulation (56). Insulin resistance impacts cardiovascular health through its effects on vascular function, macrophage accumulation, atherosclerosis development, and impairment of Ca^2+^-handling pathways in cardiomyocytes (57–59). Studies have indicated that high plasma glucose levels due to insulin resistance can provoke structural changes in vessel walls and endothelial dysfunction, leading to atherosclerotic coronary disease (CAD) and cardiovascular disease (CVD) (60, 61). Prior studies have shown that insulin receptors, such as insulin receptor-related receptor (INSRR) and insulin-like growth factor receptors (IGF1Rs), involved in the insulin signaling pathway contribute to protein kinase B (PKB/Akt) signaling, which is essential for glucose uptake and downstream phosphorylation of Ca^2+^-handling proteins (62). In case of insulin resistance, the PKB/Akt pathway becomes impaired, and cardiomyocytes are pushed towards fibrosis, dysfunction, and Ca^2+^ mishandling (57, 62).

### 4.1 Diagnostic parameters of DbCM and clinical phenotypes

Clinically, DbCM can be divided into distinct phenotypes, with dichotomy being the simplest classification. This classification includes two main phases related to left ventricle (LV), including the diastolic dysfunction stage, which is characterized by ventricular stiffness, LV hypertrophy, and increased diastolic pressure in the LV (27, 28, 63). Around 43% of individuals with T2D had left ventricular diastolic dysfunction (LVDD), 17% had HFpEF, and 22% of all individuals with diabetes had HF, making diastolic dysfunction the most prominent cardiovascular complication in diabetes (52, 64).

**Figure 2:**
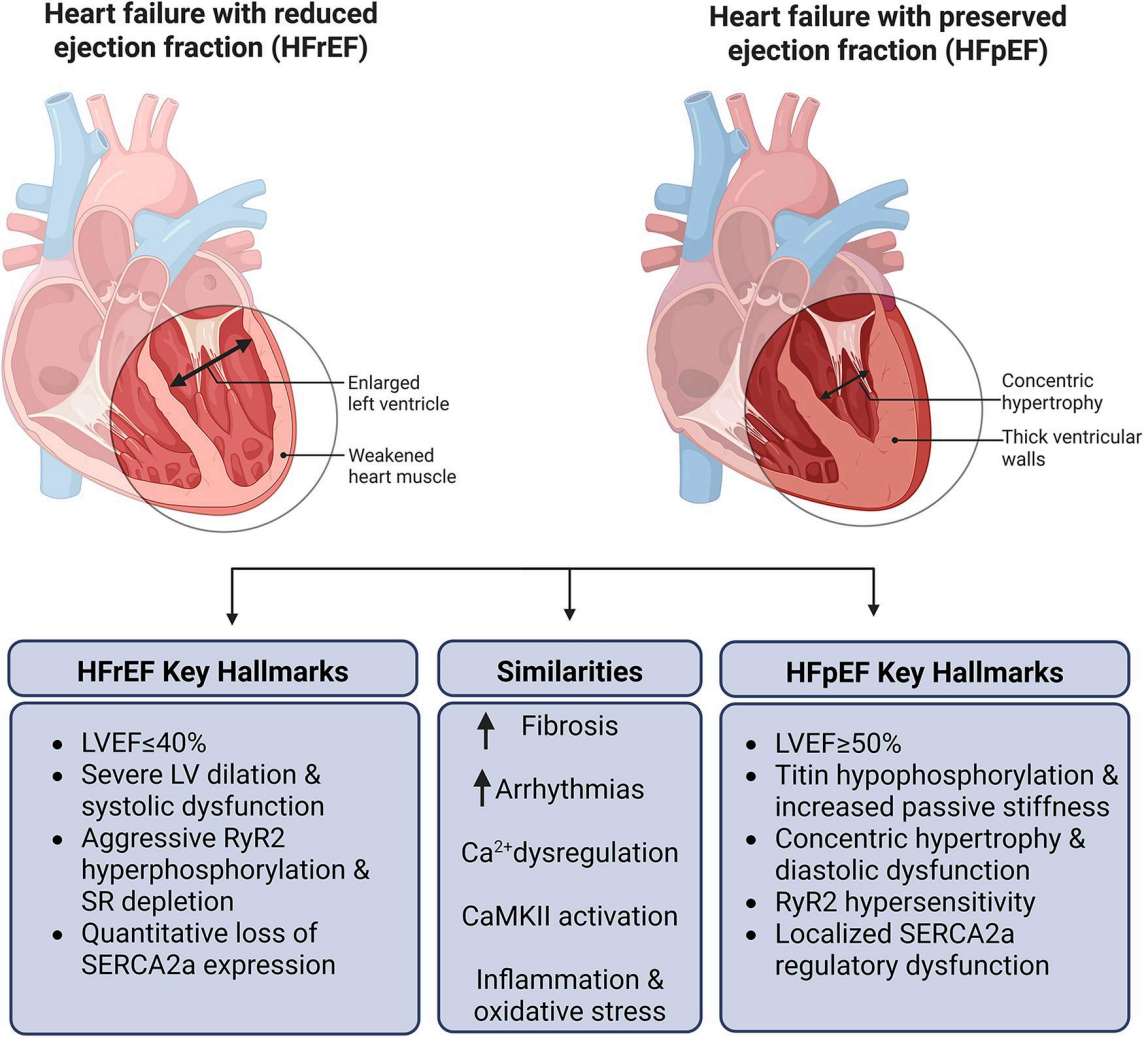
Structural and molecular hallmarks of HFrEF and HFpEF. HFrEF is characterized by LV dilation, weakened myocardium, and LVEF ≤ 40%, driven by aggressive RyR2 hyperphosphorylation, SR Ca^2^⁺ depletion, and quantitative loss of SERCA2a expression. HFpEF presents with concentric hypertrophy, thick ventricular walls, and LVEF ≥ 50%, underpinned by titin hypophosphorylation-induced passive stiffness, RyR2 hypersensitivity, and localized SERCA2a regulatory dysfunction. Both phenotypes share fibrosis, arrhythmias, Ca^2^⁺ dysregulation, CaMKII activation, and chronic inflammation and oxidative stress as common drivers. Created in BioRender. Sharma, V. (2026) https://BioRender.com/2cg1t0f.

Diastolic dysfunction in T2D diabetics with DbCM is considered a prognostic parameter (65), as proper diastolic function depends on wall thickness, fibrosis, and diastolic Ca^2^ ^+^ handling, which can be altered by chronic pathological conditions like diabetes that might lead to HFpEF (46). Myocardial fibrosis (MF) is another characteristic of worsening DbCM. MF is associated with systolic impairment, cardiac remodeling, and increased LV stiffness, which can contribute to the worsening and progression of HFpEF (46). Left ventricular hypertrophy is also related to impaired glucose tolerance (IGT) but is reversible if blood glucose levels are managed properly (66). Fibrosis, AGEs, and lipotoxicity are often associated with DbCM as causative characteristics, but the extent to which they contribute to the prognosis varies based on the type of diabetes and associated comorbidities (67). Since Ca^2+^ handling is altered during DbCM and myocardial fibrosis becomes a prognostic characteristic, it remains unclear whether myocardial fibrosis is causative or consequential of the Ca^2+^ mishandling as DbCM progresses. Figure 2 illustrates the downstream pathological effects of DbCM and Ca^2+^ dysregulation in cardiomyocytes, which leads to HFpEF and HFrEF hallmarks. As DbCM is a significant contributor of HFpEF, further studies are required to better understand the underlying characteristics of DbCM and to isolate mechanisms involved independently in the worsening of DbCM versus the consequential mechanisms. This will help determine whether fibrosis and AGEs act mechanistically or correlatively.

## 5 Molecular mechanisms underlying diabetes-induced HF

Cardiac Ca^2+^ cycling is integral for the proper maintenance of ECC (29, 48), which plays a central role in cardiomyocyte contraction under physiological conditions (68). ECC is initiated by membrane depolarization through the action potential propagation, which opens voltage-gated L-type Ca^2+^ channels (LTCC, CaV1.2), and allows Ca^2+^ influx into the cytosol (68, 69). The influx of Ca^2+^ into the cytosol triggers RyR2 opening, leading to substantial Ca^2+^ release from the SR, termed Ca^2+^-induced Ca^2+^ release (CICR) (Figure 1). The increase in cytosolic Ca^2+^ following SR release leads to contraction of the myocardium, or systole (69, 70). During relaxation, or diastole, Ca^2+^ from the cytosol is taken back up into the SR via the ATPase pump SERCA2a. Both ECC and CICR are critical for Ca^2+^ cycling under physiological conditions.

Ca^2+^ release and reuptake from the SR is highly regulated by post-translational modifications. These key Ca^2+^-handling proteins are phosphorylated by three major kinases in cardiomyocytes that include protein kinase A (PKA), Ca^2+^/calmodulin-dependent protein kinase II (CaMKII), and striated muscle preferentially expressed protein kinase (SPEG) (57, 71, 72). In addition, protein kinase B (PKB) is also critical for insulin signaling and intersects with Ca^2+^ handling. Under physiological conditions, insulin triggers PKB to phosphorylate SPEG on S2461, S2462, and T2463, leading to increased activity in mice hearts (57). Downstream, the serine/threonine kinase domain 2 (SK2) of SPEG phosphorylates SERCA2a on T484, increasing Ca^2+^ reuptake into the SR. In addition, SPEG-mediated SERCA2a phosphorylation promotes oligomerization and enhances Ca^2+^ reuptake into the SR (57). Studies have shown that both PKA- and CaMKII-mediated RyR2 hyperphosphorylation leads to Ca^2+^ leak, contributing to contractile dysfunction and HF, whereas CaMKII has shown to be the dominant kinase driving SR Ca^2+^ leaks by primarily phosphorylating RyR2 at S2814 (72, 73). The signaling pathway between PKB and SPEG is severely compromised due to insulin resistance (53). Under pathophysiological conditions, insulin resistance leads to reduced SPEG phosphorylation by PKB and further downstream effects on T484 phosphorylation of SERCA (68–70). Downregulation of SERCA2a is well documented in systolic dysfunction and end-stage HF but studies indicate preserved SERCA2a protein expression in early stages of diastolic dysfunction with a decline observed as the disease progresses, highlighting regulatory dysfunction at the structural or domain level rather than altered expression initially (74–77). Furthermore, disruption of the SPEG signaling pathway leads to diastolic Ca^2+^ leaks, a hallmark of HFpEF (46, 78, 79). This makes SPEG a potential therapeutic target, although its expression and functionality should be further studied to determine its roles as a mechanistic link between insulin signaling and Ca^2+^ handling.

### 5.1 Ca^2+^ dysregulation in diabetic cardiomyopathy

Ca^2+^ mishandling in DbCM highlights the central roles of RyR2 and SERCA2a. Studies in diabetic dogs have shown increased RyR2 phosphorylation followed by SR Ca^2+^ leak (80). Reduced Ca^2+^ removal rates in insulin-resistant mice, slower Ca^2+^ reuptake by SERCA2a along with impaired cardiomyocyte shortening and lengthening have also been observed (81, 82). Since SERCA2a-mediated Ca^2+^ reuptake is integral for cardiomyocyte relaxation, its impairment serves as a hallmark of diastolic dysfunction (83). Diabetes-induced impairment of Ca^2+^ homeostasis increases protein O-GlcNAcylation, which promotes vascular calcification, vascular stiffness, enhancing CVD risk (84). Interstitial and perivascular fibrosis are associated with DbCM, in which collagen accumulates in the extracellular matrix (ECM) and impairs normal diastolic and systolic function (85). Inflammation, insulin resistance, oxidative stress, and endothelial dysfunction all contribute to increased fibrosis during DbCM which promotes ventricular stiffness (86–88). Under diabetic conditions characterized by hyperglycemia, cardiomyocytes favor fatty acid uptake over glucose utilization as fuel, leading to mitochondrial proton leak, increased oxidative stress, and inflammation (89–91). Furthermore, AGEs start to accumulate in the heart, impairing relaxation and promoting stiffness and scarring (92, 93). In T2D, hyperinsulinemia overstimulates cardiac metabolic pathways, leading to hypertrophy, wall thickening, stiffness, and eventual beta-cell exhaustion, which contributes to insulin decline and cellular atrophy (94, 95). Obtaining cardiac biopsies to study Ca^2+^ handling in humans is challenging, but induced pluripotent stem cells (iPSCs) derived cardiomyocytes, when induced with diabetes show Ca^2+^ mishandling, reduced Ca^2+^ transients, and altered frequency (82, 95). Manganese-enhanced MRI (MEMRI) has also been used to study myocardial Ca^2+^ handling in patients with T2D and has shown impaired Ca^2+^ uptake compared with healthy individuals, although further studies are needed to establish its link with progressive HF (82). Human and animal studies have shown that Ca^2+^ mishandling contributes to DbCM progression, which often advances to HFpEF and HF (80–82, 96).

### 5.2 Structural changes, fibrosis, and ROS in HFpEF

Modification of structural proteins is a key underlying mechanism of HFpEF is hyperphosphorylation of titin, a giant (3000–4000 kDa) elastic protein that serves as a molecular spring expanding between Z-disk and M-line inside the sarcomere (40, 97, 98). Titin regulates the elasticity of the cardiomyocytes and maintains structural alignment and is a key determinant of myocardial passive stiffness and a distinguishing element compromised in HFpEF (96–98). Phosphorylation of titin is controlled by protein kinase G (PKG) mediated phosphorylation via nitric oxide-cyclic guanosine phosphate (NO-cGMP) pathway which is compromised under pathological conditions like DbCM and HFpEF leading to increased cardiomyocyte stiffness (98–100). A shift in the isoforms of titin is also observed, where N2B the less compliant and stiffer isoform is favored in HFpEF as compared to N2BA leading to titin stiffening (99). Titin-driven passive stiffness combined with SERCA2a and RyR2 mediated Ca^2+^ abnormalities build the ground for HFpEF progression and diastolic dysfunction.

HFpEF involves localized alterations in cardiomyocytes and Ca^2+^ handling proteins but there is significant cross talk amongst various cell types that promote HFpEF progression. Cardiac fibroblasts are main producers of the extracellular matrix and when activated can increase ECM deposition, myocardial stiffness and alter ECM expansion (100). Fibroblasts are also regulated by intracellular Ca^2+^ which handles collagen synthesis and myofibroblast differentiation (101). Multicellular Ca^2+^ dysregulation under pathological conditions also affects fibroblasts promoting fibrosis by activating profibrotic transcriptional pathways and favoring myofibroblast differentiation (101). Chronic oxidative stress and inflammation such as seen in DbCM and HFpEF leads to elevated ROS that activate CAMKII which further amplifies fibroblast Ca^2+^ signaling promoting myofibroblast differentiation and ECM production (39, 102, 103). This increases fibrosis by enhancing transforming growth factor beta (TGF-*β*) cytokine and ROS species release creating a self-sustaining feedback loop of myofibroblast activation, fibrosis and CAMKII activation leading to disease progression (104).

Animal models with HFpEF also demonstrate decreased oxidative phosphorylation efficiency and ATP generation during stress/exercise along with reduced mitochondrial respiratory reserves (37, 105, 106). HFpEF has been reframed as a heterogeneous disease driven by chronic and systemic inflammation (107). It incorporates comorbidities such as diabetes, hypertension, obesity and kidney disease which increase systemic inflammation through increased cytokine circulation and oxidative stress that has downstream effect (107). Since SERCA2a activity and myosin relaxation are ATP-dependent, fluctuations in ATP supply results in slow Ca^2+^ reuptake and diastolic dysfunction (108). HFpEF models also show increased ROS production by mitochondria making them a source of chronic oxidative stress which triggers the downstream CAMKII pathways and altered Ca^2+^ entry pathways (37). When these entry pathways are compromised SR Ca^2+^ release and mitochondrial Ca^2+^ sensing mismatch resulting in delayed ATP production (109). Mitochondrial Ca^2+^ signaling also serves as a regulatory mechanism for myofibroblast differentiation and when altered contributes to fibrosis and disease progression in HFpEF (110).

However, in DbCM models, it remains difficult to gauge the extent of Ca^2+^ mishandling involved in disease progression and to identify the exact disrupted pathways. This is because the major kinases including PKA, CaMKII, PKB and SPEG each have multiple downstream signaling pathways. Thus, further studies in animal models and human cohorts are needed to clarify the exact mechanisms involved in DbCM. Differences in sex, species, and age across studies make it difficult to translate findings to human models, which contributes to the current knowledge gap.

## 6 Animal models

Insulin resistance and DbCM have been replicated in rodent models to study disease progression. The most common animal models used to recapitulate diabetes are those placed on high-fat diets (HFD) combined with streptozotocin (STZ) injections, which destroy pancreatic cells to simulate insulin deficiency and induce hyperglycemia (111, 112). Mice aged 4–8-weeks are typically placed on a HFD for 5–8 weeks and receive STZ near the end of the regimen to induce diabetes and insulin resistance, leading to cardiac impairment, hypertrophy, diastolic dysfunction, and phenotypic DbCM (111, 112). Other common animal models used to study diabetes-induced DbCM include genetically altered *ob/ob* and *db/db* mice. The *ob/ob* mouse carries a mutation in the leptin gene and is therefore unable to produce leptin to regulate satiety, whereas the *db/db* mouse carries a mutation in the leptin receptor inhibiting leptin signaling (113). Both *ob/ob* and *db/db* mice exhibit cardiac impairment and structural remodeling, making them useful for studying systolic and diastolic dysfunction, as well as LV hypertrophy (114, 115). In 2019, a mouse model fed both 60% HFD and N*ω*-nitro-L-arginine methyl ester (L-NAME) was shown to fully recapitulate diabetes-induced HFpEF (116). Use of this nitric oxide synthetase (NOS) inhibitor in combination with HFD induced structural and functional changes observed in HFpEF, a hallmark of diastolic dysfunction. Diastolic function has also been shown to be impaired in mice with DbCM because of SERCA2a dysfunction and increased SR Ca^2+^ leak through RyR2 (96). PKB deletion, which mimics insulin resistance in humans and impairs insulin-mediated PKB-SPEG phosphorylation as well as Ca^2+^ reuptake through SERCA2a in mice, has also been shown to compromise cardiac function (57). Mice fed sucrose (SU)-based diets, compared with mice fed starch (ST)-based diets, exhibited SERCA2a impairment and slower Ca^2+^ reuptake, although SERCA2a protein levels remained unchanged, suggesting cardiomyocyte dysfunction in insulin-resistant mice (81, 82). Dogs fed a HFD also demonstrated increased PKA-mediated phosphorylation of S2809 on RyR2, which promotes RyR2 leak leading to contractile dysfunction and arrhythmias (80). Mice with systemic insulin resistance and impaired glucose tolerance, mirroring diabetes in humans, showed impaired insulin-mediated PKB activation and disrupted SPEG and SERCA2a phosphorylation pathways. In isolated cardiomyocytes from these mice, Ca^2+^ transients were reduced, whereas full duration at half maximum (FDHM) and time constants (tau, τ) were increased, indicating impaired Ca^2+^ handling and reuptake into the SR (116). Another study using HFD to induce HFpEF in mice showed that isolated cardiomyocytes collected from these mice had elevated diastolic Ca^2^ ^+^ levels, increased Ca^2+^ transients, and frequency-dependent slowing of Ca^2+^ reuptake, suggesting Ca^2+^ mishandling and SERCA2a impairment (43). Although these studies have been conducted to strategically examine Ca^2+^ handling and its associated pathways, they lack in encapsulating the true translational relevance to humans. HFpEF animal models remain insufficient translationally because not all HFpEF models created are equal as they only capture one or two phenotypes of HFpEF arising from comorbidities such as diabetes, hypertension, and obesity (117–120). HFpEF is a network of failures incorporating Ca^2+^ dysregulation, fibrosis, inflammation, and endothelial dysfunction all creating a loop that feeds towards the same purpose of progressing HFpEF (119, 121). Therapies targeting one or the other mechanisms are limited in their efficacy as they lack to constitute the remaining fronts being affected by this disease.

### 6.1 AAV vectors in cardiac gene therapy

**Figure 3:**
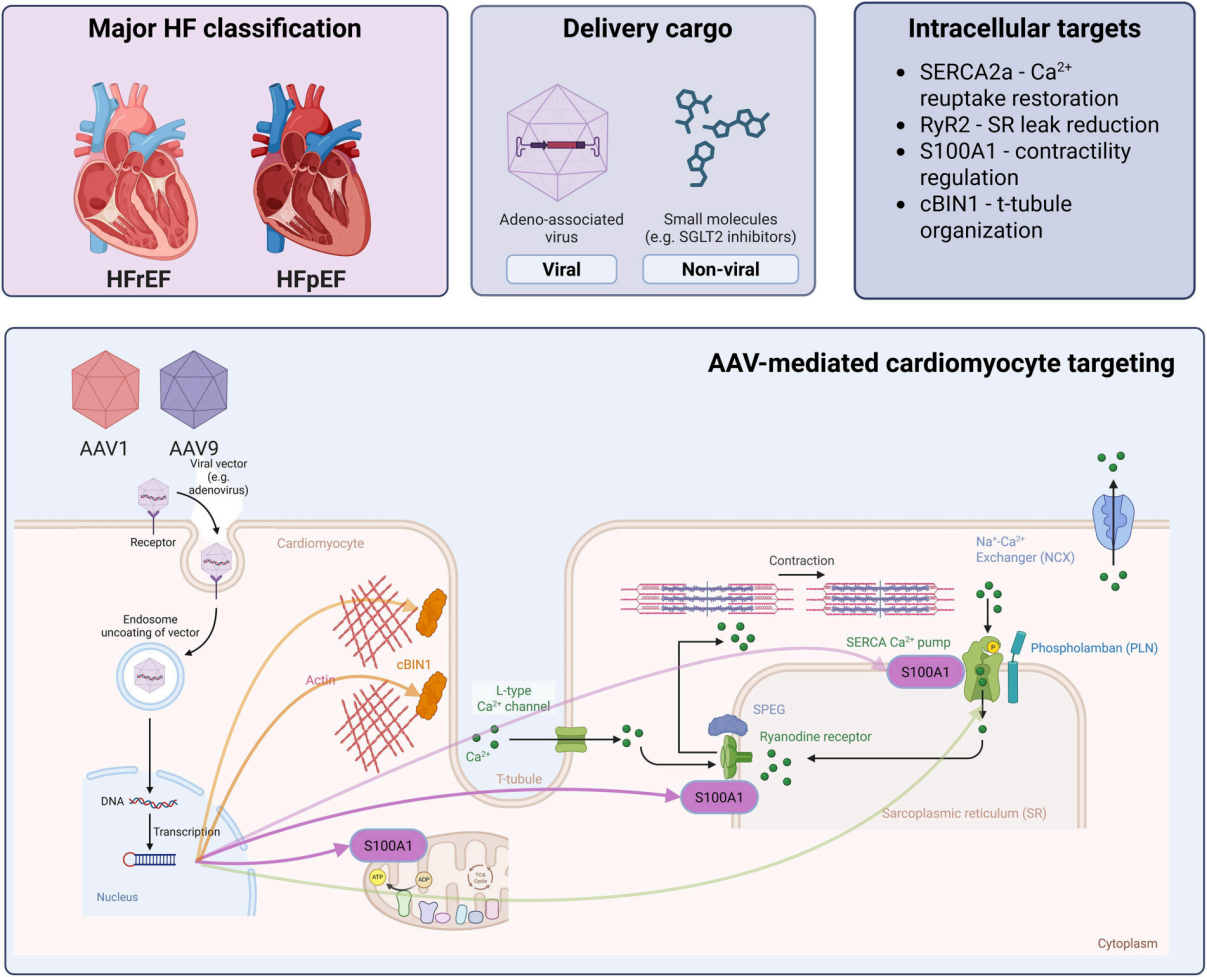
AAV-mediated gene therapy strategies for cardiac Ca^2+^ restoration in HF. The top panels illustrate the two target HF phenotypes (HFrEF and HFpEF) and delivery modalities, including viral vectors such as AAV1 and AAV9, and non-viral small molecules such as SGLT2 inhibitors. The lower panel depicts AAV-mediated cardiomyocyte transduction, in which the vector enters via receptor-mediated endocytosis, undergoes endosomal uncoating, and enables nuclear transcription of the therapeutic transgene. Key intracellular targets such as SERCA2a, RyR2, S100A1, andcBIN1, are highlighted within the Ca^2+^ handling machinery of the sarcoplasmic reticulum and t-tubule system. Created in BioRender. Sharma, V. (2026) https://BioRender.com/4g1me2g.

Recently, adeno-associated viruses (AAVs) have increasingly been used to reverse pathological CVD phenotypes through restoration of Ca^2+^-handling proteins (122–126) (Figure 3) (Table 1). HF mouse models have been used to evaluate AAV-mediated restoration of dwarf open reading frame (DWORF), which enhances SERCA2a activity and increases Ca^2+^ reuptake (125). AAV-mediated reintroduction of *DWORF* restored Ca^2+^ cycling, cardiac function, energy supply, and SERCA2a activity by directly targeting the SERCA2a-PLN axis, which restored the Ca^2+^ reuptake (125). In another study using myosin-binding protein C 3 (*MYBPC3)-*deficient murine models, AAV was used to restore *MYBPC3* that is involved in cross-bridge cycling and contractility that maintains sarcomere integrity. AAV-MYBPC3 mediated restoration resulted in reversal of cardiac hypertrophy, improved cardiac function, and reduction in systolic dysfunction (122). The efficacy of AAV-*S100A1* was also evaluated preclinically in porcine HF models (123). S100A1 regulates cardiac contractility and is depleted during HF. Restoration of *S100A1* using AAV9 led to normalized Ca^2+^ cycling, SR Ca^2+^ handling, sustained transgenic expression, and improved energy balance (123). Canine models with surgery-induced progressive dilated cardiomyopathy, HF, and LVEF <40% received endocardial injections of AAV9-*cBIN1* (124). Cardiac bridging integrator 1 (cBIN1) is a protein that downregulates during HF, and is responsible for organizing t-tubules and Ca^2+^-handling machinery (81). Canines administered with AAV9-*cBIN1* showed improved LV function, t-tubule morphology, endocardial voltage, and LV chamber size compared with untreated control animals (81). Preclinical trials in both small and large animal models have shown that restoration of Ca^2+^ handling improves HF symptoms and cardiac function. AAV-mediated therapies have been actively used in animal models for more than a decade, but their long-term efficacy remains insufficiently studied. Although improvements have been reported in animal models, these effects have been modest and lower than expected, which, combined with severity of disease progression raises questions regarding treatment efficacy. Optimistically, the range of proteins and pathways being tested through AAV therapies provides multiple potential targets for restoring LV function and slowing disease progression.

**Table 1:** Summary of AAV and non-viral therapeutic approaches targeting cardiovascular disease and diabetic cardiomyopathy.

Vector	Target Gene	Disease model	Species/model	Year Published	Key findings	Reference
AAV9	*S100A1* Transgene	Post-ischemic HF	Porcine	2011	Increased Contractility	(85)
AAV1	*SERCA2a*	Ischemic, nonischemic cardiomyopathy and advanced HF	Human patients (randomized, double-blind, placebo-controlled Phase 2 clinical trial)	2011	SR Ca^2^⁺ handling improved, contractility improved	(92)
AAV1	*SERCA2a*	Advanced HF (NYHA III/IV)	Human patients (multicenter, randomized, double-blind, placebo controlled Phase 2b clinical trial)	2015	No significant improvement LV function	(110)
Non-viral plasmid DNA	SDF-1/*CXCL12*	Chronic ischemic HF with LV dysfunction	Human patients (Phase 2 trial randomized, double-blind, placebo-controlled)	2015	Improved LV remodeling	(40)
AAV8	UCn2	Insulin resistance and impaired glucose homeostasis	Murine	2016	Improved insulin sensitivity, glucose tolerance, and whole-body glucose disposal	(108)
Ad5-hAC6	*AC6*	HFrEF	Human patients, (randomized, double blind, placebo-controlled Phase 2 Clinical trial)	2016	Increased LVEF at 4 weeks (but not sustained at 12 weeks)	(94)
AAV8	*UCn2 and UCn3*	Chronic HF	Murine	2018	Improved LV systolic function, UCn2 produced greater inotropic benefit	(107)
OGT and OGA	*OGA*	DbCM (T1D & T2D)	Murine (*db/db*)	2018	Improved systolic function and attenuated fibrosis	(37)
OGT and OGA	*OGA*	DbCM (T1D & T2D)	Murine (transgenic, *db/db*)	2022	O-GlcNAcylation improved diastolic function, Ca^2^⁺ handling, and fibrosis	(109)
AAV9	*cBIN1*	DbCM	Murine (*db/db)*	2023	Reversed structural and functional hallmarks of HFpEF-like DbCM	(100)
AAV9	*cBIN1*	Chronic nonischemic HF (dilated cardiomyopathy)	Mini-pigs	2024	Reversed LV dilation and improved global systolic function	(101)
AAV9	*DWORF*	HF with mitochondrial dysfunction (post ischemic)	Murine	2025	Restored mitochondrial respiration Improved LV systolic function	(81)
AAV9	*cBIN1*	Chronic HF	Canine Model	2025	Enhanced LV systolic function and reversed HF remodeling	(80)
AAV2i8	*I-1c*	Non-ischemic cardiomyopathy	Humans (Phase 1 clinical trial)	2025	Improved LVEF and exercise capacity in higher-dose groups	(90)
AAV2i8	*I-1c*	HFrEF	Human Patients (Phase 2, randomized, double-blind placebo controlled)	2025	Improved LV function, clinical status, and reduced HF events	(120)
Finerenone	Non-steroidal MR	Chronic kidney disease with T2D	Human patients (randomized, double-blind, placebo-controlled Phase 3 trial)	2021	Reduced CV events and renal decline, lowered hyperkalemia risk	(127)
Finerenone	Non-steroidal MR	HFpEF or HFmrEF	Humans	2025	Reduced CV deaths and HF events	(128)
Finerenone	Non-steroidal MR	HF with improved EF	Humans	2025	Reduced HF hospitalization and continued benefit in HF with improved EF	(129)
Empagliflozin	SGLT2 inhibitor	HFpEF	Human patients (randomized, double-blind, placebo-controlled Phase 3 trial)	2021	Reduced CV deaths and HF hospitalization	(130)
Dapagliflozin	SGLT2 inhibitor	HFpEF or HFmrEF	Human patients (randomized, double-blind, placebo-controlled Phase 3 trial)	2022	Reduced CV deaths and HF worsening	(131)

## 7 Therapeutic approaches

In 2024 alone, global medical expenses related to diabetes surpassed USD 1 trillion, a substantial increase from USD 966 billion spent in 2021, approximately 50% of which was attributable to diabetes-related complications^5^. This escalating economic burden highlights the limitations of current therapeutic approaches and calls for innovative preventative strategies. To mediate the situation AAVs are widely used as viral vectors for gene transfer and modification. These recombinant AAV vectors are non-pathogenic and have the ability to transduce with a helper virus for coinfection (132–134). For gene therapy, the gene of interest is typically packaged into a transfer plasmid between inverted terminal repeats (ITRs) together with a tissue-specific promoter (135, 136). These transfer plasmids are then introduced into HEK293 cell lines together with rep/cap and helper plasmids for AAV capsid production and subsequent delivery to the target tissue (135–137). AAV-mediated therapies have increasingly been evaluated for CVDs in studies using animal models and human clinical trials to assess their therapeutic potential in cardiac disease (138–143). Since AAVs can persist inside cells as episomes and insertional mutagenesis during standard therapeutic use is rare, they represent a reliable platform for gene delivery (144, 145). Their small capsid size facilitates passage through capillaries, even dispersion throughout the myocardium, and retention within the highly vascular cardiac environment (146). Many cardiac-specific targets are sufficiently small to fit within the AAV genome, including *SERCA2a, PLN, S100A1*, miRNAs, shRNAs, and Ca^2+^-handling modulators, all of which are typically < 4 kb (123, 143, 147, 148). These viruses comprise multiple serotypes, ranging from AAV1 to AAV12, with distinct tissue tropisms. AAV1, AAV6, AAV8, and AAV9 have strong cardiac tropism with AAV9 outperforming other serotypes due to its efficiency in crossing vascular endothelium and cardiomyocyte transduction after intravenous administration. These serotypes have been used to deliver genes of interest, including *SERCA2a, MYBPC3,* and *S100A1*, to cardiac tissue through intravenous or intracoronary administration in animal models of CVD (122, 123, 137, 149–151).

### 7.1 Clinical evidence supporting key Ca^2+^-handling protein restoration effects

AAV-based cardiac therapies have advanced to first-in-human trial of AB-1002, an AAV vector encoding protein phosphatase-1 inhibitor (I-1c), which liberates SERCA2a inhibition by PLN and increases Ca^2+^ reuptake (126). Improvements in LVEF have been observed alongside persistent transgene expression in cardiac tissue (126). A phase 1 cardiotropic trial (NCT04179643) in patients with LVEF of 15%–30% showed considerable improvements in ejection fraction, exercise capacity, and cardiac function following intracoronary and intramyocardial AAV delivery, followed by sustained transgene expression over several months (138).

These findings are further supported by studies in small and large animal models, including rodents, dogs, and pigs, in which AAV6, AAV8, and AAV9 were used to restore genes like *SERCA2a, S100A1, cardiac bridging integrator 1 (cBIN1),* resulting in phenotypic reversal and improved survival (123, 124, 140, 152). In these clinical trials, AAV met acceptable safety profiles across all dosage levels and resulted in cardiac improvements as well as sustained long-term gene expression. These dose-dependent improvements in humans and animal models with HF suggest that AAV-based therapies promote early phenotypic reversal and improved therapeutic efficacy. Most safety concerns surrounding AAVs arise from high vector doses, which can lead to toxicities. Clinically, high AAV doses can lead to acute liver injury, hepatotoxicity, thrombocytopenia and extreme innate and adaptive immune responses that can be fatal (153, 154). Humans naturally have neutralizing antibodies (NAbs) against common AAV serotypes due to environmental exposure, these NAbs reduce AAV transduction and initiate immune clearance compromising the efficacy of AAV mediated treatment options (155, 156). AAV use in Duchenne muscular dystrophy (DMD) and spinal muscular atrophy (SMA) has resulted in dose-linked toxicities, including thrombotic microangiopathy, hemolytic anemia, kidney injuries, fatal liver injury, and neurotoxicity (157–160). One AAV9-mediated mini-dystrophin phase 1b trial by Pfizer reported 2 cases of myocarditis, one of which was fatal, most likely because of severe inflammation, whereas in another trial using AAVrh74, one patient developed myocarditis that was controlled with steroids (161, 162). Most toxicities are dose-dependent and more likely to develop with systemic delivery. However, in CVDs, this risk may be mitigated with efficient capsids and strong promoters ensuring localized/targeted effects.

### 7.2 Potential for reversing diabetic cardiomyopathy

Studies in *db/db* mice with induced HFpEF have shown that AAV9-*cBIN1* normalized Ca^2+^ handling, insulin-mediated glucose uptake in cardiomyocytes, exercise tolerance, and hyperglycemia (151). AAV8 vectors encoding *Urocortin 2 (UCn2)* and *Urocortin 3 (UCn3)* have been shown to improve LV systolic and diastolic function through improved Ca^2+^ handling mediated by increased SERCA2a expression (127). In mice with HF, UCn2 has also been shown to reduce fasting glucose and increase glucose disposal and insulin sensitivity, making it a suitable option for treating diabetic cardiomyopathy (107). Another study showed that male mice with HFD-induced insulin resistance received a one-time AAV8-*UCn2* injection, which normalized blood glucose levels, increased glucose uptake plus insulin sensitivity, and reduced plasma insulin (128). The effect lasted for months, suggesting that AAV8-facilitated *UCn2* delivery may have potential to reverse insulin resistance-related diabetic cardiomyopathy (108). Murine models with induced diabetes and LV diastolic dysfunction were treated with rAAV6-*OGA*, in which O-GlcNAcase (OGA) facilitates removal of the O-GlcNAc sugar tag from proteins (129, 130). In diabetic conditions, excess O-GlcNAc due to hyperglycemia hinders diastolic function and increases fibrosis plus cardiac remodeling (130). Mice treated with rAAV6-*OGA* showed improved LV diastolic function, decreased cardiac remodeling, and dose-dependent improvement in cardiac function in mice with diabetes-induced cardiomyopathy (37, 109). Restoration of Ca^2+^ handling through the reintroduction of microproteins such as DWORF, which enhance SERCA2a activity, has also been shown to reverse HF symptoms in mice with transverse aortic constriction TAC-induced HF. AAV-mediated DWORF overexpression helped alleviate contractile dysfunction, normalize Ca^2+^ cycling through SERCA2a, and reduce cardiac remodeling (125). The CUPID phase 2 trial also demonstrated that AAV-mediated *SERCA2a* delivery improved cardiac function and reduced hospitalizations in patients with advanced HF, although phase 2b did not yield similar results or improvements in high-risk patients with HFrEF (92, 110). Phase 2b failed to reproduce significant improvements as expected and observed in the prior phase, no significant LVEF improvement or clinical outcomes were seen which led to early termination of the trial (110). AAV1 being used in phase 2b has pre-existing NAbs that significantly reduce vector uptake and transduction, reducing treatment efficacy. Additionally, patients with severe HFrEF exhibit extensive cardiac remodeling that further limits reversibility and the approach is more intended to slow disease progression (110, 116). AAV-mediated *SERCA2a* gene therapies have been tested in HF, with improvements observed to an extent in systolic and diastolic function, as well as Ca^2+^ cycling but approaches integrating modified capsids with increased transduction and immune-modulating strategies are required to get dependable and reproducible results. For HFpEF-based trials, Medera announced results in May 2025 for its ongoing trial, “Modulation of SERCA2a of Intra-myocytic Calcium Trafficking in Heart Failure with Preserved Ejection Fraction” (MUSIC-HFpEF),the first-in-human phase 1/2a clinical trial using AAV. Patients reported no liver enzyme abnormalities, and AAV1-*SERCA2a* intracoronary infusions were well tolerated. The study reported improved pulmonary capillary wedge pressure (PCWP) and exercise tolerance in patients, but half of the patients had stabilized NT-proBNP levels, whereas the other half had worsened NT-proBNP levels; NT-proBNP is a marker of cardiac stress^6^ (163).

Recent trials involving two major drug classes, sodium–glucose cotransporter 2 (SGLT2) inhibitors and finerenone a non-steroidal mineralocorticoid receptor (MR) antagonist have shown positive outcomes in reducing CVDs related hospitalizations and deaths amongst patients with HFpEF and other comorbidities (164–168). SGLT2 inhibitors block the SGLT2 transporter in the proximal renal tube which reduces glucose and sodium reabsorption improving glycemic control, reducing inflammation and fibrosis as well as lowering cardiac preload and afterload. Finerenone on the other hand, selectively blocks mineralocorticoid receptor signaling reducing aldosterone-induced inflammation and fibrosis in cardiac and renal tissues (169–174). Both these drugs have anti-fibrotic and anti-inflammatory properties that help slow down renal decline and HF related hospitalization in patients suffering from comorbidities such as diabetes, hypertension, obesity and HFpEF (164, 175–177).

## 8 Advantages and limitations of AAV-mediated gene therapies

AAV-mediated gene therapies have been pursued as potential therapies for HF and CVDs for decades. Table 1 summarizes several novel gene therapies in preclinical and clinical stages, but the success rate of these trials has been modest. Challenges associated with AAV use include poor myocardial transduction, large fractions of vectors accumulating in the liver rather than the heart, and the need for high AAV doses to accommodate large proteins, which can induce immunogenic reactions that slow down the translational process (178, 179). AAV1, AAV6, and AAV9 have been shown to have the greatest cardiac tropism, but species-specific differences can compromise efficacy when moving from preclinical to clinical trials (160). Promoters are required to ensure tissue specificity, but some are too weak to support therapeutic expression, whereas others lack sufficient cardiac specificity, making promoter selection a barrier to safe translation (180). Size constraints pose challenges for larger proteins and cardiac modulators such as SPEG (11 kb), myosin heavy chain 7 (MYH7; 23 kb), and RyR2, which cannot fit within a single AAV genome at full length (181–183). Due to immune memory, repeated dosing is not an ideal option, and single doses can be rendered ineffective primarily through pre-existing antibodies that block AAV uptake or clear it out before reaching the heart (179). Small animals are easier to target in terms of cardiac tissue specificity and gene transduction than humans, in whom dosage requirements and tropism alter outcomes substantially, rendering many successful animal preclinical trials ineffective at the clinical stage (184).

It is therefore essential to focus on treatments targeting Ca^2+^ regulation as it is well integrated into the mechanisms that drive disease progression in both HFpEF and HFrEF. Despite these setbacks, translational studies remain ongoing, and emerging clinical trials continue to evaluate AAV-mediated therapies for HF and CVDs (138, 185). Efforts are underway to overcome size constraints and immuno-toxicities using mini-genes, micro-gene constructs, trans-splicing vectors, and dual AAV systems to target larger proteins and improve therapeutic efficacy (178, 186–189).

## Footnotes

^1^Diabetes

^2^IDF Diabetes Atlas 2025 | Global Diabetes Data & Insights

^3^National Diabetes Statistics Report - United States Diabetes Surveillance System

^4^National Diabetes Statistics Report - United States Diabetes Surveillance System

^5^IDF Diabetes Atlas 2025 | Global Diabetes Data & Insights

^6^Study Details | NCT06061549 | Modulation of SERCA2a of Intra-myocytic Calcium Trafficking in Heart Failure With Preserved Ejection Fraction | ClinicalTrials.gov

## Glossary

AAV: adeno-associated viruses
AC6: adenylyl cyclase type 6
ADA: American Diabetes Association
AGE: advanced glycation end-products
Ca^2+^: calcium
CAD: atherosclerotic coronary disease
CAMKII: Ca^2+^/calmodulin-dependent protein kinase II
cBIN1: cardiac bridging integrator 1
CDC: Centers for Disease Control and Prevention
CICR: Ca^2+^-induced Ca^2+^ release
CUPID: calcium upregulation by percutaneous administration of gene therapy in cardiac disease
CVD: cardiovascular disease
CXCL12: C-X-C motif chemokine ligand 12
DbCM: diabetes-induced cardiomyopathy
DMD: Duchenne muscular dystrophy
DWORF: dwarf open reading frame
ECC: excitation-contraction coupling
ECM: extracellular matrix
EF: ejection fraction
FDHM: full duration at half maximum
FPG: fasting plasma glucose
GLUT4: glucose transporter type 4
GTT: glucose tolerance test
hAC6: human adenylyl cyclase type 6
HbA1c: glycated hemoglobin
HEK293: human embryonic kidney 293 cells
HF: heart failure
HFD: high-fat diets
HFmrEF: heart failure with mildly reduced ejection fraction
HFpEF: heart failure with preserved ejection fraction
HFrEF: heart failure with reduced ejection fraction
I-1c: protein phosphatase-1 inhibitor-1
IDF: International Diabetes Federation
IFG: impaired fasting glucose
IGF1Rs: insulin-like growth factor receptors
IGT: impaired glucose tolerance
INSRR: insulin receptor-related receptor
iPSC: induced pluripotent stem cells
ITR: inverted terminal repeats
L-NAME: N*ω*-nitro-L-arginine methyl ester
LTCC: L-type Ca^2+^ channels
LVDD: left ventricular diastolic dysfunction
LVEF: left ventricular ejection fraction
LV: left ventricular
MEMRI: manganese-enhanced MRI
MF: myocardial fibrosis
miRNA: microRNA
MR: mineralocorticoid receptor
MUSIC-HFpEF: modulation of SERCA2a of intra-myocytic calcium trafficking in heart failure with preserved ejection fraction
MYBPC3: myosin-binding protein C3
MYH7: myosin heavy chain 7
NO-cGMP: nitric oxide-cyclic guanosine phosphate
NOS: nitric oxide synthetase
NT-proBNP: N-terminal pro-B-type natriuretic peptide
NYHA III/IV: New York Heart Association functional classes III and IV
OGA: O-GlcNAcase
OGT: O-GlcNAc transferase
PCWP: pulmonary capillary wedge pressure
PKA: protein kinase A
PKB or PKB/Akt: protein kinase B
PLN: phospholamban
PP1: protein phosphatase 1 inhibitor-1
RAGE: receptor for advanced glycation end-products
ROS: redox or reactive oxygen species
RyR2: ryanodine receptor type 2
SDF-1: stromal cell–derived factor 1
SERCA2a: sarcoplasmic reticulum Ca^2+^-ATPase
SGLT2: sodium–glucose cotransporter 2
shRNA: short hairpin RNA
SK2: serine/threonine kinase domain 2
SMA: spinal muscular atrophy
SPEG: striated muscle preferentially expressed protein kinase
SR: sarcoplasmic reticulum
STZ: streptozotocin
SU/ST: sucrose-/starch-based diets
T1D: type I diabetes
T2D: type 2 diabetes
TAC: transverse aortic constriction
TGF-*β*: transforming growth factor beta
UCn2: urocortin 2
UCn3: urocortin 3
WHO: World Health Organization.
